# Targeting Post-Translational Regulation of p53 in Colorectal Cancer by Exploiting Vulnerabilities in the p53-MDM2 Axis

**DOI:** 10.3390/cancers14010219

**Published:** 2022-01-03

**Authors:** Chunwei W. Lai, Cindy Xie, Jean-Pierre Raufman, Guofeng Xie

**Affiliations:** 1 Department of Medicine, Division of Gastroenterology & Hepatology, University of Maryland School of Medicine, Baltimore, MD 21201, USA; walter.lai@nih.gov (C.W.L.); CindyXie2016@gmail.com (C.X.); jraufman@som.umaryland.edu (J.-P.R.); 2 VA Maryland Healthcare System, Baltimore, MD 21201, USA; 3 Marlene and Stewart Greenebaum Comprehensive Cancer Center, University of Maryland School of Medicine, Baltimore, MD 21201, USA; 4 Department of Biochemistry and Molecular Biology, University of Maryland School of Medicine, Baltimore, MD 21201, USA

**Keywords:** degradation, colorectal cancer, p53, MDM2, negative feedback

## Abstract

**Simple Summary:**

p53, a critical tumor suppressor, is commonly mutated in neoplasia, including colorectal cancer. To devise anti-cancer strategies targeting p53, it is crucial to understand the myriad cell-specific regulatory mechanisms in the p53 signaling pathway, and how these same regulatory mechanisms may be evaded by p53 mutants. This review focuses on colorectal cancer and considers the regulatory mechanisms underlying the actions of wild type p53 protein, emphasizing discoveries made in the last decade. We focus on the role of mouse double minute 2 homolog (MDM2), which modulates p53 protein levels by targeting p53 for protein degradation; other MDM2-independent mechanisms are also discussed. These regulatory mechanisms are further examined in the context of p53 missense mutants, which can evade canonical regulation. Lastly, we consider potential strategies for therapeutic targeting of p53 mutant-bearing cancers in preclinical testing or early-phase clinical trials.

**Abstract:**

The role played by the key tumor suppressor gene p53 and the implications of p53 mutations for the development and progression of neoplasia continue to expand. This review focuses on colorectal cancer and the regulators of p53 expression and activity identified over the past decade. These newly recognized regulatory mechanisms include (1) direct regulation of mouse double minute 2 homolog (MDM2), an E3 ubiquitin-protein ligase; (2) modulation of the MDM2-p53 interaction; (3) MDM2-independent p53 degradation; and (4) inhibition of p53 nuclear translocation. We positioned these regulatory mechanisms in the context of p53 missense mutations, which not only evade canonical p53 degradation machinery but also exhibit gain-of-function phenotypes that enhance tumor survival and metastasis. Lastly, we discuss current and potential therapeutic strategies directed against p53 mutant-bearing tumors.

## 1. Introduction

Worldwide, colorectal cancer (CRC) remains in the top five most prevalent and lethal cancers [1]. In sporadic CRC, the most common form, loss of p53 function is a critical step in the adenoma-to-adenocarcinoma transition; p53 mutations are detected in 55–60% of non-hypermutated and ~20% of hypermutated CRC [2,3]. As p53 controls important cellular functions, including cell cycle checkpoints and senescence, under normal physiological circumstances its expression is tightly regulated. However, the contribution of p53 mutation to tumorigenesis is more than simply a result of loss of p53 function. Some p53 mutations exhibit gain-of-function properties that ‘supercharge’ tumor growth and metastasis. Adding to this complexity is the observation that p53 network regulation varies between cell types. To devise effective therapeutic strategies to target p53, it is crucial to gain a comprehensive understanding of p53 dysregulation. In this review, we examined post-translational regulatory mechanisms underlying p53 homeostasis, focusing on discoveries made in the last decade and shedding light on how p53 mutants escape regulatory controls in the context of CRC. Unless otherwise specified, the information reported here is based on CRC laboratory models and clinical data. We conclude by discussing gaps in knowledge and how what we know now can be applied to develop therapeutic strategies directed against p53 mutants. To aid the reader, we summarize new modulators of the p53 pathway in Table A1. 

### 1.1. Post-Translational Regulation of p53 Expression

Commonly referred to as the “guardian of genomes”, p53 controls the cell cycle, senescence, and apoptosis, although non-canonical functions in oxidative phosphorylation, epithelial–mesenchymal transformation, and modulation of the tumor microenvironment are reported [4]. The p53 protein is a 393-amino acid tumor suppressor divided into several domains: an N-terminal transactivation domain, a central DNA-binding domain, a tetramerization domain, and a lysine-rich C-terminal domain. Tetramerization enables p53 to bind to downstream promoters. The lysine-rich C-terminal domain is the site of key post-translational modifications including ubiquitination. In response to genotoxic stress, p53 can be activated via phosphorylation at Ser-15 by ATM and CHK; activated p53 forms tetramers to bind target genes [4]. Intranuclear p53 levels determine cell fate and are regulated by the combination of post-translational modification, proteasomal degradation, and nuclear translocation. More recently, p53 levels were found to oscillate throughout the cell cycle without cell arrest under non-stress conditions, a feature thought to maintain its high sensitivity to cellular DNA damage [5,6]. Transcriptional regulation of p53 expression, beyond the scope of the present review, was reviewed elsewhere [7].

### 1.2. Murine Double Minute 2 (MDM2) Regulation

**Figure 1:**
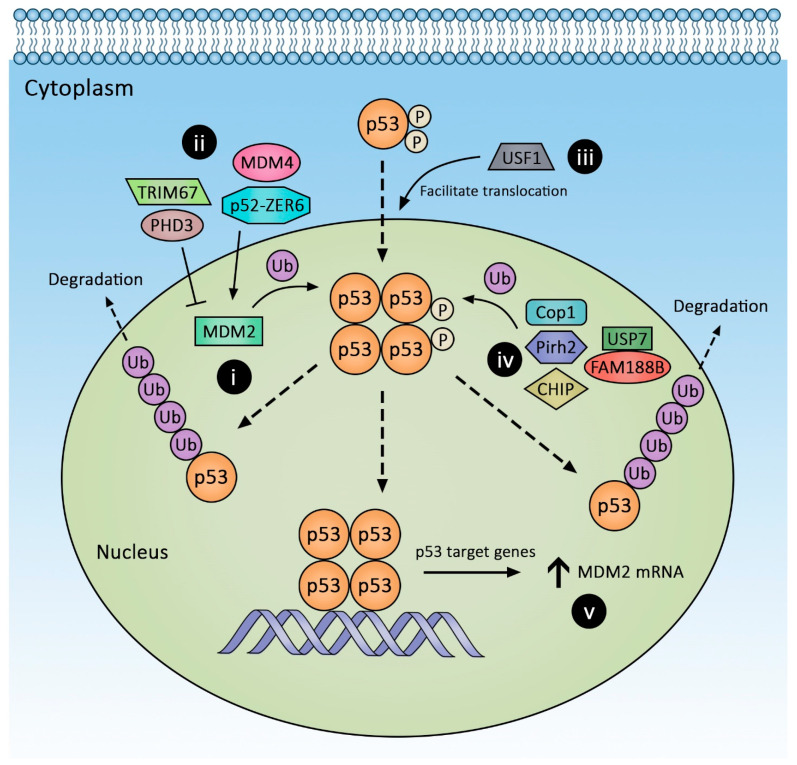
Key molecules involved in post-translational regulation of wild-type p53. Post translational regulation of p53 involves several distinct mechanisms, including (i) direct interaction with mouse double minute 2 homolog (MDM2), which stimulates proteasomal degradation; (ii) PHD3, TRIM67, MDM4, and ZER-p52 modulate MDM2 function; (iii) USF1 facilitates translocation of the MDM2/p53 complex into the cytoplasm for degradation; (iv) MDM2-independent p53 degradation via CHIP, Pirh2, COP1, and the USP7-FAM188B complex; and (v) MDM2 transcriptional upregulation by p53 via a negative feedback loop. Decreased p53 levels promote CRC progression.

E3 ligases mediate polyubiquitination of a substrate and target it for proteasomal degradation. The most important E3 ligase regulating p53 levels is MDM2. The non-redundancy of this property of MDM2 is evidenced by observing pan-p53 activation in multiple tissues of MDM2 knockout (KO) mice, which results in diffuse apoptosis and embryonic lethality [8]. The MDM2 KO phenotype can only be rescued by concomitant p53 KO. To study the function of MDM2 in adult animals, investigators introduced a tunable p53 expression system (p53ER^TAM^). The important functional domains of MDM2 include the N-terminus domain, which binds to both the N- and C-terminus regions of p53 [9,10,11], and the C-terminus domain, which contains a RING finger motif that carries out the E3 ligase function and a central zinc finger/acidic domain that serves as an allosteric binding site that modulates p53-MDM2 interaction [12,13]. As shown in Figure 1, MDM2 regulates p53 function in at least two ways: At high MDM2 levels, MDM2 polyubiquitinates p53 and targets it for proteasomal degradation, whereas at low MDM2 levels, p53 is mono-ubiquitinated, resulting in its cytoplasmic translocation. The latter action inhibits p53’s transactivation function via spatial exclusion [14,15]. MDM2 expression is induced by p53 itself; myriad mechanisms regulating MDM2 activity are discussed below. 

### 1.3. MDM2/4 Homodimers

MDM4 (or MDMX) shares homology with MDM2, including a RING domain. MDM4/X was initially thought to have overlapping function with MDM2, but using kidney and breast cancer cell lines, Wang et al. [16] showed MDM4 actually modulated MDM2. In the DNA damage response, MDM4 binds MDM2, thereby enhancing MDM2’s ability to poly- rather that mono-ubiquitinate p53. In homeostasis, MDM4 appears to facilitate translocation of the MDM2/p53 complex into the cytoplasm for proteasomal degradation. However, following genotoxic exposure in a human CRC cell line, MDM4 dissociates from the MDM2/4 complex and instead binds to and stabilizes HIPK2. HIPK2 then induces p53Ser46 phosphorylation, which induces p53-mediated apoptosis [17]. With less severe genotoxic exposure, MDM2 degrades HIPK2 and p53 remains inactive [18]. 

### 1.4. Modulation of MDM2 Functions via Its Acidic Domain

The regulatory property of the zinc finger/acidic domain in MDM2 was first recognized from a series of MDM2 aberrant splicing mutants bearing wild-type p53 [19]. This central domain binds to basic peptides such as p14^ARF^ (p19^ARF^ in mice) [20], E2F1 [21], and ribosomal proteins (L5, L11, L23) [13], thereby inhibiting MDM2-induced p53 degradation. Interestingly, p19ARF and ribosomal proteins (RP) inhibit MDM2 function via different mechanisms. Using a missense mutant MDM2^C305F^ that loses the ability to bind RP but retains binding to p19ARF and its E3 ligase function in an APC-null mouse background, it was shown that c-Myc upregulation due to loss of APC increases RP L5 and L11 expression and predisposes animals to CRC [22].

Prolyl hydroxylase 3 (PHD3) is a novel MDM2 regulator (Figure 1(ii)). PHD3, originally known for targeting hydroxylating hypoxia-inducible factor 1a (HIF-1a) for degradation, has additional substrates including MDM2. Xu et al. [23] showed that PHD3 overexpression inhibited MDM2 activity by binding to its zinc finger/acidic domain and increased p53 protein levels. Notably, this is independent of PHD3 hydroxylase activity. PHD3 overexpression results in p53-dependent downregulation of CRC stem cell properties in vitro; however, its in vivo effects require further investigation.

### 1.5. Acidic Domain-Independent Regulators of MDM2-p53 Interaction

TRIM67 is a new tripartite motif (TRIM) family protein capable of modulating p53 levels (Figure 1(ii)). TRIM protein members possess a RING domain that, among other cellular functions, permits the protein to act as an E3 ligase. The tumor suppressive roles of TRIM proteins were previously hypothesized but unproven. To identify potential TRIM protein(s) with tumor suppressive properties, Wang et al. [24] undertook a systemic search for epigenetic modification of TRIM genes using the TCGA database. They discovered the promoter of TRIM67 was uniquely hypermethylated and suppression of TRIMP67 expression correlates with poor CRC outcomes. Additionally, TRIM67 levels are lower in tumors than adjacent nontumor tissues. In two CRC-induction models, *Apc^Min/+^* and azoxymethane (AOM)-treated mice, homozygous loss of TRIM67 predisposed animals to tumorigenesis. Using p53 truncation mutants, these investigators found TRIM67 binds to the C-terminus of p53 and disrupts MDM2-p53 binding. In a positive-feedback loop, p53 also increases TRIM67 expression. Overexpressing TRIM67 does not stabilize p53 mutants ((mutp53) R273H, P309S, S241F), and mutp53s do not increase TRIM67 expression. 

ZER6 is another recently discovered oncogene with two isoforms, p52 and p71 via alternative splicing. Initially recognized as a regulator of p21, a downstream effector of p53, ZER6 overexpression is associated with better CRC outcomes. Further investigation using human HCT116 CRC cells revealed that the ZER6-p52 isoform suppresses p21 expression by downregulating p53 at the translational level. ZER6-p52 binds to both p53 and MDM2 (Figure 1(ii)), thereby stabilizing the p53-MDM2 complex and resulting in p53 degradation [25]. Nutlin-3, a small molecular inhibitor of MDM2 that stabilizes wild-type p53, outcompetes the enhancing effect of ZER6-p52 on p53-MDM2 complex [25,26]. It is not known if ZER6-p52 preserves modulating capacity against mutp53s.

Another means of modulating the p53-MDM2 interaction is competition for the p53 binding site on MDM2. Beta-lactamase-like protein (LACTB), a novel modulator, acts in this manner. Related to bacterial penicillin-binding B, LACTB is ubiquitously expressed in mammalian tissue cytoplasm and mitochondria. Early studies focused primarily on the effects of LACTB in the inflammatory response; its link to CRC, neuroblastomas, and breast cancer only emerged in recent years. In CRC, LACTB downregulation is associated with a poor prognosis [27]. LACTB suppresses tumor growth by competing with MDM2 for binding at the C-terminus of p53 in HCT116 and HCT8 CRC cell lines. Loss of LACTB expression in cancer is generally due to epigenetic silencing rather somatic mutation. Notably, loss of LACTB does not impact CRC in the context of p53 null or p53-mutant phenotypes [27].

Upstream Stimulating Factor-1 (USF1) also emerged as a novel regulator of the p53 pathway (Figure 1(iii)). A ubiquitous cytoplastic protein with a bHLH domain, USF1 was previously considered a stress sensor and transcriptional regulator. Using B16 melanoma cell line and mouse fibroblasts, Bouafia et al. [28] were the first to show USF1 binds to p53 during genotoxic stress and prevents the MDM2-p53 interaction, thereby stabilizing p53 and permitting its nuclear translocation. Co-immunoprecipitation assays revealed that USF1 and MDM2 interact separately with p53, suggesting mutually exclusive interactions. Elevated USF1 expression levels are associated with a poor CRC prognosis [29,30]. Unlike other stress-induced transcription factors that regulate p53 stability (e.g., YY1, ATF3, TAFII31), USF1 is expressed constitutively and may play a more prominent role in regulating p53 during genotoxic stress [28]. Precisely how mutp53s interact with USF1 remains undetermined. 

### 1.6. Modulation of MDM2 Protein Stability

MDM2 self-ubiquitination can regulate its levels; MDM2 de-ubiquitination prolongs its half-life and consequently reduces p53 protein levels. USP7 (previously known as HAUSP), a deubiquitinase, interacts with MDM2 [31]. USP7 also binds p53 directly; Brooks et al. used a H1299 cell culture model to show USP7 binding to p53 or MDM2 is mutually exclusive [31]. FAM188B binds to USP7 and destabilizes the USP7-p53 interaction, thereby promoting p53 ubiquitination and degradation (Figure 1(iv)). Deletion of FAM188B decreases CRC cell (HCT-116, SW620, and HT-29) xenograft growth, and, in the TCGA dataset, FAM188B expression levels are inversely correlated with CRC survival [32]. How FAM188B expression is regulated remains unknown. Targeting USP7 in cancer has been considered. However, it is important to understand that while MDM2 KO can be rescued by p53 ablation, USP7 KO cannot be rescued the same way, suggesting USP7 regulates additional critical substrates [33]. Small molecule USP7 inhibitors alone fail to inhibit p53 degradation fully and, for tumor suppression, require synergy with a MDM2 inhibitor [34]. Regardless, these findings reveal the therapeutic potential of small-molecule mimics of FAM188B. It remains unclear if USP7 can engage mutp53s. A new USP7 inhibitor with sub-nanomolar potency and specificity against multiple cancer cell lines appears ineffective against tumors harboring p53 hotspot mutants [35].

### 1.7. Transcriptional and Post-Transcriptional Regulation of MDM2

Activated p53 binds to the *MDM2* promoter and drives MDM2 expression, allowing for negative feedback of the p53 pathway. As reviewed by Zhao et al. [36], estrogen receptors, Ras, and MYCN are additional transcriptional regulators of p53. MDM2 is also regulated by microRNAs [37]. Mdm2 possesses an exceptionally long 3’-UTR that can be the target of many miRNAs. Two miRNA clusters—miR-194-2-192 and miR-215-194-1—were the first shown to regulate CRC cell MDM2 mRNA [38]. Additional miRNAs capable of regulating MDM2 expression in CRC and other cancers include miRNA-143 [39], 145 [40], 605-5p [41], 17-3p [42], 193a-5p, and 146a-5p [43].

### 1.8. Novel MDM2 Regulator with an Undefined Mechanism

Other novel regulators of MDM2 are emerging, but their molecular mechanisms are yet to be fully dissected. An example is Inhibitor of Differentiation and DNA-binding protein 1 (ID1), a HLH family protein that regulates cell differentiation. A correlation between ID1 and MDM2 expression was first reported in esophageal cancer [44]. Recently, ID1 was found to be in the downstream signal cascade of Leukemia Inhibitory Factor (LIF), overexpressed in CRC [45]. LIF induces STAT3 activation, which drives ID1 expression; higher expression of the ID1 axis increases MDM2 levels, thereby promoting p53 degradation, although how ID1 increases MDM2 protein requires further clarification. 

### 1.9. Other p53-Degrading E3 Ligases: Beyond MDM2

#### 1.9.1. Co-Chaperone Carboxyl Terminus Hsp70/90 Interacting Protein (CHIP)

The network of Hsp70, Hsp90, and their co-chaperones regulate p53 stability and activity via the protein folding quality-control mechanism. Hsp70 promotes p53 unfolding and proteasomal degradation, specifically by interacting with the DNA-binding domain. In contrast, Hsp90 promotes DNA-binding domain folding and enhances p53 DNA-binding capability [46,47,48]. Several mutp53s are associated with Hsp90, resulting in enhanced stability [49,50]. CHIP is a tetratricopeptide repeat-containing protein with E3 ligase activity that also interacts with Hsp70 and Hsp90 C-terminal domains. Comparison between cells harboring different mutp53s showed that conformational mutp53 (R175H) exhibits prolonged association with Hsp70, targeting mutp53 for proteasomal degradation, whereas wild-type p53 only binds transiently to Hsp70 [50]. CHIP overexpression enhances degradation of both forms of p53, whereas CHIP KO has opposite effects. This “triage” role of CHIP [51] is largely controlled by Hsp90. Hsp90 binds to the MDM2-p53-Hsp70-CHIP complex, thereby suppressing CHIP activity and preventing p53 degradation until substrates achieve a proper conformation [50]. Some mutp53s are terminally misfolded, leading to their prolonged association with Hsp90 and extending their stability. Additional modulatory factors such as Hop and BAG1 that may contribute to Hsp90-CHIP interaction were reviewed elsewhere [52]. 

More recently, CHIP was found capable of degrading mutp53 via autophagy in transgenic models, a mechanism that may not apply to properly folded mutp53 variants including R173H and R243W [53]. CHIP also ubiquitinates Mortalin-2, a Hsp70 family protein overexpressed in cancer that suppresses p53 nuclear translocation [54]. UBSXN2A, a ubiquitin-like protein, was shown to promote mortalin-2 degradation by CHIP and, in turn, liberate p53 from Mortalin-2 to translocate into the nucleus and induce growth arrest and apoptosis in CRC cells [54,55]. 

#### 1.9.2. Pirh2

Pirh2 is another p53-specific E3 ligase that facilitates p53 degradation. In contrast to MDM2, Pirh2 binds to aa 100-300 of p53 [56]. Pirh2’s contribution to p53 degradation appears less important as, unlike MDM2 KO, Pirh2 KO is not embryonic lethal. Recently, Pirh2 was shown to contribute to the growth of hormone-driven pro-oncogenesis in colonic epithelial cells by reducing p53 levels [57].

#### 1.9.3. COP1

COP1, another E3 ligase thought to mediate p53 degradation, was reviewed elsewhere [58]. There are no reports linking COP1 expression to CRC tumorigenesis and its expression does not appear to have prognostic value in CRC (gene data available from v20.proteinatlas.org) [29,59]. 

### 1.10. Post-Translational p53 Modifications 

Since p53 was discovered in 1979, a wealth of knowledge has accrued regarding how post-translational modifications such as acetylation, ubiquitination, phosphorylation, methylation, sumoylation, and neddylation determine p53 half-life and activity [4]. The p53 N-terminal domain contains multiple phosphorylation sites; p53 phosphorylation results in its activation and binding to downstream promoters, as well as resistance to MDM2-mediated degradation. In contrast, the C-terminal domain is enriched with post-translational modification sites for ubiquitination, acetylation, and methylation, setting up competition between different regulatory mechanisms. For example, lysines in the p53 C-terminal domain are not only subject to ubiquitination but also to other post-translational modifications including acetylation. The p53 acetylation promotes tumor suppression without activation of apoptosis [60]. The p300/CBP acetylates the C-terminal domain, preventing MDM2 binding to p53 [61,62]. In an opposing effect, at least in CRC cancer cells, Jumonji domain-containing 6 (JMJD6) competes to hydroxylate the same lysine-rich domain in the p53 C-terminal domain, thereby preventing acetylation of these same residues [63]. Hydroxylation promotes p53 interaction with MDM4/X, which recruits MDM2 to facilitate p53 degradation. Acetylation of p53 can also be reversed by deacetylases such as the sirtuin family. A nucleus-localizing sirtuin family protein, SIRT1 directly deacetylates p53 protein and decreases p53 transcriptional function [64,65]; however, p53 also regulates SIRT1 expression via the miR34a-5p axis in CRC [66]. 

While ubiquitination is commonly associated with proteasomal degradation, it can also modulate p53 function in other cellular processes [67]. For example, Msl2, WWP1, Ubc13, and E4F1 mediate degradation-independent ubiquitination, typically outside the MDM2 binding sites. Interestingly, ubiquitination at some of these degradation-independent sites protects p53 against MDM2-mediated degradation. 

Recently, a regulatory role for tumor necrosis factor receptor-associated factor 6 (TRAF6) was identified in the p53 pathway beyond its classic role in immunoregulation. Zhang et al. [68] showed that TRAF6, a non-conventional E3 ligase, inhibited p53-mediated tumor suppression during genotoxic stress via an intriguing, two-step mechanism: (1) TRAF6 suppresses cytosolic p53 mitochondrial translocation via K63 ubiquitination thereby suppressing apoptosis and (2) TRAF6 ubiquitinates p53 at K24, thereby facilitating p53 engagement with p300, p53 acetylation, and anti-apoptotic upregulation of p21. High TRAF6 expression correlates with a poor prognosis following chemoradiation therapy for CRC [68]. 

### 1.11. The p53 Regulation by Subcellular Localization

p53 must be physically present in the nucleus to bind to target gene promoters and activate tumor suppressive programing. Spatial exclusion from the nucleus suppresses downstream p53 signaling. In the absence of cell stress, p53 localizes to the cytoplasm by associating with anchor proteins [69]. Another molecular mechanism that achieves spatial exclusion is exporting p53 out of the nucleus, a strategy employed by MDM2 in addition to its E3 ligase function. 

TRIM28 (ATDC) also regulates p53 function via spatial exclusion. TRIM28, which sequesters p53 in the cytoplasm without degradation [70], is implicated in growth hormone-mediated nuclear p53 suppression. In addition to growth hormone-associated suppression of Pirh2-mediated p53 degradation, TRIM28 creates a “permissive” oncogenic environment in the colon [57]. Sho et al. showed in CRC cells that Tip60 inhibition can contribute to the anti-apoptotic effect of TRIM28 [71]. 

## 2. Regulation of Gain-of-Function p53 Mutant Expression 

Loss of p53 function is a hallmark of cancer development, specifically in the CRC adenoma-to-carcinoma transition. Many p53 mutations result in loss of functional proteins (74–80%) [72], but some result in proteins with gain of function (GOF), i.e., with novel activities [73,74]. 

The importance of GOF phenotypes first became apparent when mutp53 expression in a p53-null cell line displayed oncogenic properties [75]. Later, individuals with germline p53 missense mutations (Li–Fraumeni Syndrome) were found to have earlier cancer onset and shorter survival compared to those with germline p53 null mutants [76,77]; these findings were supported by similar observations in animal models [78]. Oncogenic phenotypes can be abolished if mutp53 expression is suppressed or ablated in vivo [79]. These GOF mutations enhance cell proliferation, increase colony formation, and promote cell invasion and migration, angiogenesis, and chromatin remodeling. 

GOF mutants often arise from accumulation of missense mutations in tumor cells [80,81,82]. Malignant transformation is believed to be triggered by DNA damage, at least in the R175 mutants [80]. Similar to wild-type p53, MDM2 appears capable of regulating mutp53, as evidenced by enhanced mutp53 levels when mutp53-bearing animals are crossed with MDM2 KO mice [82]. The assumption is that mutp53 cannot trigger the same downstream pathway as wild-type p53, including induction of MDM2; consequently, low MDM2 abundance results in increased mutp53 stability. Indeed, mutp53 mice subjected to ROS, radiation, and DNA-damaging chemotherapy stabilized mutp53 protein, resulting in earlier tumor onset and shorter survival [83]. A recent report demonstrates that some mutp53s can bind to the central zinc finger/acidic domain of MDM2, thereby directly inhibiting its E3 ligase activity—another mechanism prolonging mutp53 half-life [84].

Although p53 GOF mutants have survival advantages, loss of heterozygosity (LOH) permits GOF mutants to reach their full malignant potential. Approximately 93% of human cancers lose wild-type p53 through loss of heterozygosity [85], and its mechanistic importance was best seen in stabilizing nuclear localization of mutp53 [85,86,87]. Using a R270H mutant in murine intestinal organoid models, Nakayama et al. demonstrated that wild-type p53 suppressed the mutp53 phenotype; LOH enhances the metastatic capability of tumors by increasing mutp53 stability and nuclear localization [88]. 

Studies tend to generalize mutp53s, but they are functionally diverse and could have varying prognostic values. In a compilation of survival analysis of several cancer types, including CRC, from a combined dataset of The Cancer Genome Atlas (TCGA) and MSKCC bladder cancer dataset (JCO, 2013), totaling 2916 cases, Xu et al. [89] examined how individual p53 hotspot mutations affect outcomes in human cancer. Survival outcomes appear similar between structural, contact, and nonsense mutations. Surprisingly, survival outcome also did not differ significantly between wild-type p53 and mutp53. When the survival outcomes of individual GOF mutations were compared to nonsense mutation using Kaplan–Meier survival analyses, patients with R248 and R282 mutations-bearing tumors had a significantly worse prognosis. These findings were validated in a separate dataset. To identify the mechanism contributing to the worse prognosis associated with R248 and R282 mutations, Xu et al. performed gene enrichment analysis on the CRC dataset in TCGA (n = 224 [3]). They showed that, unlike the two intensely studied p53 mutations, R175H and R273H, R248Q/W and R282W mutations shared 52 commonly enriched genes: The shared pathways center around cytochrome p450 CYP3A4, an important gene for drug metabolism. CYP3A4 expressions were elevated in tumors bearing R248Q/W and R282W p53 mutations; enhanced tumor resistance to chemotherapy is likely to explain worse survival outcomes. 

## 3. Therapeutic Strategies against p53 Mutants

**Figure 2:**
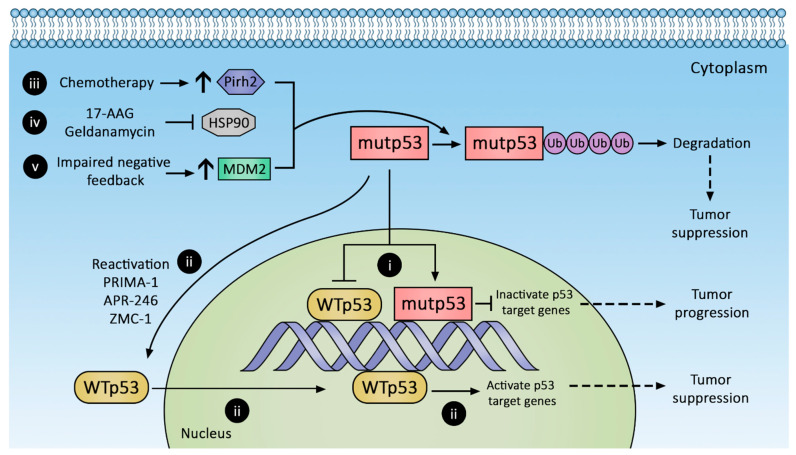
Therapeutic strategies against p53 mutants (mutp53). Mutp53 promotes CRC progression, whereas reactivation and decreased mutp53 suppress CRC. Mutp53 inactivation of p53 target genes (i) can be reversed by (ii) reactivating wild-type p53 with PRIMA-1, APR-246, and ZMC-1 or augmenting mutp53 degradation with (iii) chemotherapy to increase Pirh2 expression, (iv) inhibiting HSP90, and (v) increasing MDM2 expression.

Tumor dependence (addiction) on mutp53 GOF led to targeted therapy to suppress mutp53 expression by protein degradation (Figure 2), transcriptional suppression, and conversion to wild-type p53 phenotype. Novel immunotherapies leveraging neoantigens in mutp53 are also emerging. These approaches are discussed below.

### 3.1. Promoting mutp53 Protein Degradation

#### 3.1.1. Increasing MDM2 Expression

As reviewed above, mutp53 accumulation has been attributed to impaired negative feedback between MDM2 and mutp53. MDM2 can bind and ubiquitinate mutp53, although with decreased efficiency compared to wild-type p53 [90,91]. Augmenting MDM2 expression is believed to degrade mutp53 and suppress tumor growth, but a recent study by Yang et al. revealed that mutp53 (R175H, G245S, R248Q, R273H, D281G) inhibits MDM2 activity [84]. In addition, desired depletion of mutp53 in tumors or generalized depletion of wild-type p53 in normal tissues are difficult to achieve. The other concern is that a global increase in MDM2 expression may have an untoward effect on normal tissues and inadvertently promote tumorigenesis. The feasibility of this strategy has yet to be tested in the clinic. 

#### 3.1.2. Stabilizing MDM2-mutp53 Complex

Theoretically, small molecules can be designed to target the abovementioned MDM2 modulators via an agonist or antagonist mechanism. However, few, if any, are in preclinical testing, and to our knowledge, none have been evaluated for colorectal cancer.

#### 3.1.3. Hsp90 Inhibition

Mutp53 subtypes exploit protein folding machinery such as Hsp90 to increase their half-life, and the inadvertent activation of stress responses to misfolded proteins also enhances malignant cell survival. Of the six p53 mutation “hot spots” (R175, G245, R248, R249, R273, R282), nearly all demonstrate prolonged engagement with the Hsp90 complex compared to wild-type p53, suggesting they are prone to protein misfolding [50]. Geldanamycin (Figure 2(iv)) is a first-generation Hsp90 inhibitor that shows great efficacy in diverting p53 conformation mutants toward the Hsp70-CHIP complex to promote their degradation [48]. However, despite antitumor efficacy, geldanamycin is plagued with adverse effects, most notably hepatotoxicity [92]. Geldanamycin analogues with reduced toxicity have been engineered (e.g., 17-AAG), but they have low oral bioavailability [93].

Newer generations of non-geldanamycin-derived Hsp90 inhibitors emerged in the last decade. Ganetespib, an early agent in this class, is 50-fold more potent than 17-AAG at inhibiting Hsp90 [79], with improved solubility and reduced toxicity [94]. TAS-116, another novel agent, is orally active and selective against cytoplasmic Hsp90. Although TAS-116 monotherapy shows limited antitumor effect [95], TAS-116 plus nivolumab, an immune checkpoint inhibitor, demonstrates an augmented treatment response against microsatellite-stable (MSS) CRC compared to TAS-116 monotherapy [96]. 

HDAC6 regulates Hsp90 expression and its associated protein machinery; inhibition of HDAC6 hyperacetylates Hsp90, thereby abolishing Hsp90 binding to its partners [97]. SAHA, a HDAC inhibitor explored as an alternative method of inhibiting Hsp90, demonstrates preferential toxicity to mutp53-expressing cells [98]. 

#### 3.1.4. Pirh2 Upregulation

Another approach to promote mutp53 degradation is with arsenic trioxide, which induces Pirh2 expression and promotes proteasomal degradation of mutp53s [99,100] (Figure 2(iii)). Mutp53s susceptible to Pirh2-mediated regulation include R175H, R248W, H179Y/R282W, and R273H. Arsenic trioxide is commonly evaluated as part of combination therapy, e.g., with Gemtuzumab ozogamicin in previously untreated acute promyelocytic leukemia (NCT01409161) or with itraconazole in advanced basal cell cancer (NCT02699723). In 2010, a phase 1 trial of 5-fluorouracil, leucovorin, and arsenic trioxide for refractory CRC established a safe dose [101], but no phase 2 trial is currently registered on clinicaltrials.gov. 

### 3.2. Conversion of mutp53 to Wild-Type p53

Selective secondary mutations in the C-terminal domain of mutp53 can restore proper protein folding in the transactivation domain and DNA-binding domains, thereby restoring wild-type p53 characteristics [102,103,104]. Small synthetic peptides with similar allosteric stabilization properties targeting the C-terminal domain of mutp53 can also restore the wild-type p53 phenotype. A search for small molecules with the abovementioned properties yielded PRIMA-1 and its analog PRIMA-1^Met^ (also known as APR-246). PRIMA-1 and APR-246 (Figure 2(ii)) are prodrugs of the active compound methylene quinuclidinone (MQ), which forms covalent bonds with cysteines in mutp53, restoring wild-type-like sequence-specific binding and inducing tumor apoptosis [105,106,107]. Their clinical efficacy remains unknown. A highly anticipated phase 1b/2 clinical trial combining APR-246 with carboplatin for the treatment of recurrent high-grade ovarian cancer (NCT02098343) was completed in April 2019, but no results have been reported. Other trials evaluating combined APR-246 and azacitadine for various hematologic malignancies (NCT03931291, NCT04214860, NCT03745716, NCT03588078) are ongoing, with at least one in phase 3.

Zinc metalochepaeron-1 (ZMC-1; NSC319726), another compound capable of reactivating mutp53 (Figure 2(ii)), was discovered in a drug screen using an NCI60 tumor cell line panel [108]. ZMC-1 promotes zinc binding with mutp53, thereby stabilizing its folding and restoring transcriptional activity. Its reactivation efficacy is limited to the R175H mutation, currently the third most common p53 hot spot mutation; however, other defective zinc-binding mutants such as C176, C242, C238, H179, and M237 may also be restored by ZMC-1 [109].

### 3.3. Restoring Downstream p53 Pathways

Sidestepping the dysfunctional mutp53 and directly remediating p53 downstream signaling is another attractive strategy. The p63/p73 belongs to pathways downstream of p53 and shares structural and functional similarity to wild-type p53, capable of tumor suppression. However, p63/p73 does not interact with wild-type p53. In contrast, some mutp53s (such as R270H) do bind to and exert dominant negative effects on p63/p73 [110]. A small molecule RETRA was shown to inhibit mutp53-p73 complex formation, thereby restoring the tumor suppressive function of native p73 in mutp53-bearing cells while sparing normal cells [111]. 

Activating Transcription Factor 3 (ATF3) is another druggable target that can restore p53 downstream pathway. ATF3, a stress-induced transcription factor, is known to compete with MDM2 for p53, thus preventing ubiquitination of wild-type p53 [112]. However, more recently, ATF3 was also shown to prevent mutp53 (R175H and R273H) from binding to p63 in CRC cell lines, thereby restoring the p53 downstream pathway [113]. Inhibiting Hsp90 restores ATF3 expression and confers tumor suppression [114]. Antitumor actions of Hsp90 inhibition could stem from the synergetic effects of increased mutp53 degradation and ATF3 upregulation. Patulin, a fungal toxin, can also increase ATF3 expression in colorectal cancer cell lines and induce cell apoptosis, possibly via reactive oxygen species signaling [115]. 

### 3.4. Targeting p53 Mutant Neoantigen for Immunotherapy

Other emerging strategies against mutp53s-bearing tumors, albeit ‘out of the box’ compared with the abovementioned strategies, aim to exploit the immunogenicity of mutp53s. The hypothesis is that once antibodies and/or tumor-infiltrating lymphocytes (TIL) recognize p53 neoantigens on the MHC complex, either direct or indirect immune response can be elicited to eliminate mutp53-expressing cells. Although anti-mutp53 autoantibodies can be detected in the serum of individuals with cancer, high antibody titers correlate with poor prognosis instead of improved outcome [116,117,118,119]. The lack of protective efficacy in anti-mutp53 autoantibody was attributed to improper antibody targeting to the amino and carboxyl termini of mutp53 instead of the central mutation hotspot [118]. Using a large phage library screen, Hsiue et al. [120] identified an antibody that uniquely recognizes mutp53 (R175H) but not wild-type p53. They cloned this specific antigen binding site into one end of a novel bi-specific antibody for drug targeting, and incorporated antigen specificity for T cell-CD3 complex on the other end of the antibody to elicit host lymphocytic immune response. In vivo efficacy of this bi-specific antibody was evaluated against a mutp53-bearing myeloma cell line (KMS26) implanted in mice with a reconstituted human immune system. The bi-specific antibody suppressed tumor growth but expectedly requires the presence of human T cells. This exciting finding awaits confirmation in clinical trials. Along the same design rationale, in the hope of identifying TCR specific for mutp53 neoantigen and also to expand TIL for cell-based therapy, an ongoing NCI study [121] seeks to identify tumor-infiltrating lymphocytes that recognize mutp53 neoantigens. The field of immunotherapy holds great promise as it specifically directs cytotoxicity at cancers. Since hotspot mutations change p53 structure, it is likely other hotspot mutations will exhibit unique antigens that can be targeted immunologically. Potential limitations include the high cost of drug development and testing, side effects resulting from immune activation, and the likely constraints in individuals with germline p53 mutations.

## 4. Conclusions and Future Directions

Despite nearly five decades of p53 research, new insights in the role of mutp53 in tumor pathogenesis continue to emerge. Novel p53 regulators contribute to the increasingly complex p53 regulatory network, but some are context-dependent and not all contribute to CRC progression. Different mutp53s evade regulation via various mechanisms; some demonstrate gain-of-function phenotypes that augment tumor aggressiveness. Identifying strategies to circumvent mutp53 expression is the goal of p53-targeted therapy. This is especially critical in tumors demonstrating addiction to mutp53 GOF. Other promising approaches include exploiting mutp53 neoantigens for immunotherapy. However, many key issues in the regulation of wild-type and mutp53s in the context of CRC remain unanswered. For example, what are the predominant regulatory mechanisms for each p53 mutant? What methods can predict them? Sporadic CRC commonly harbors multiple mutations. How do mutp53s synergize with other mutated pathways? Understanding the synergism may identify additional targets for therapeutic intervention. Lastly, leveraging next-generation sequencing, such as single-cell RNAseq, of individual CRC may help tailor therapies targeting the p53–MDM2 axis. 

## Appendix A

**Table A1:** Newly identified modulators of the p53 pathway.

Gene	Mechanism	Effect on Wild-Type p53 Levels
*HIPK2*	When bound by MDM4, phosphorylates/activates p53 [17]	Increase
*PHD3*	Inhibits MDM2 via binding to the acidic domain [23]	Increase
*TRIM67*	Disrupts p53-MDM2 binding [24]	Increase
*ZER6-p52*	Stabilizes p53-MDM2 complex [25]	Decrease
*LACTB*	Competes with MDM2 for p53 binding [27]	Increase
*USF1*	Competes with MDM2 for p53 binding [28]	Increase
*FAM188B*	Binds to USP7 and inhibits its p53 deubiquitination [32]	Decrease
*LIF/STAT3/ID1-axis*	Increases MDM2 expression [45]	Decrease
*UBSXN2*	Increases Mortalin-2 degradation, thereby liberating p53 [54]	Increase
*JMJD6*	Increases p53-MDM4-MDM2 interaction [63]	Decrease
*TRAF6*	Suppresses mitochondrial translocation of p53, and enhances p53 acetylation [68]	Decrease
*TRIM28*	Inhibits p53 nuclear translocation [70]	Decrease
miR-194-2-192 cluster, miR-215-194-1 cluster, miR-143, miR-145, miR-605-5p, miR-17-3b, miR-193a-5p, miR-146a-5p	Increase MDM2 mRNA degradation [38]	Increase
